# Epidemiological profile of health consultations during the Mozambique 9^th^ National Cultural Festival, August 2016

**DOI:** 10.11604/pamj.2019.33.52.15534

**Published:** 2019-05-23

**Authors:** Judite Monteiro Braga, Lucas Nhantumbo, António Nhambomba, Esmeralda Cossa, Crescêncio Nhabomba, Tomas Dimas, Daniele Nunes, Cristolde Salomão, José Paulo Langa, Cynthia Baltazar, Jonas Brant

**Affiliations:** 1 Mozambique Field Epidemiology Training Program (Moz FELTP), Mozambique; 2 Instituto Nacional de Saúde (INS), Maputo, Mozambique; 3 Department of Epidemiology, National Directorate of Public Health, Ministry of Health, Maputo, Mozambique; 4 Centro de Investigação Operacional de Beira (CIOB), INS, Beira, Mozambique; 5 Associação Brasileira de Profissionais de Epidemiologia de Campo (ProEpi), Brazil

**Keywords:** Mozambique, culture, festival, epidemiological, health consultations, mass gathering

## Abstract

**Introduction:**

The use of mass gatherings as spaces to practice health surveillance has been growing in recent years. In Mozambique, the 9th National Festival of Culture in 2016 was selected for this practice. A specific public health surveillance system to facilitate rapid detection of outbreaks and other health-related events was implemented for this event with real time data collection and analysis

**Methods:**

A descriptive epidemiological evaluation of all the health consultations that occurred in fixed posts prepared for the event was conducted. The data were collected through electronic mobile system (tablets) in real time, with the aid of a form designed for this purpose and sent directly to the incident command system (ICS)

**Results:**

During the event, a total of 355 patients were assisted, 52.3% were female, 87.0% were from Beira city and the artists were the group that most frequently sought health care at 59.4%. The largest number of visits took place on the third day (36.4%). People over 45 years of age were the age group that most frequently sought health care (30.8%). The main provisional diagnoses of those who were attended to during the festival was arterial hypertension (20.3%), followed by febrile syndrome (19.0%), with falls being the most frequent causes of trauma during the festival (60.0%).

**Conclusion:**

The system of monitoring in real time using mobile technologies proved to be efficient for the monitoring of the main health events during the mass gatherings. This profile of health consultations encourages the health sector to plan strategies and actions geared to the reality of care for this type of event.

## Introduction

Mass gatherings, defined as “an organized or unplanned event where the number of people attending is sufficient to strain the planning and response resources of the community, state or nation hosting the even during a specific period of time” [1, 2]. Constitute a public health challenge, since it may incur risks to health with rapid spread of infectious diseases “imported” from participants' places of origin or by the illness of persons not immune or “adapted” to local diseases [1, 3-5]. The greatest health risks during the mass gathering events include diseases related to various environmental factors, diseases transmitted by food and/or water, communicable diseases, accidents or trauma [1], the increase in consumption of drugs and alcohol, stings and bites from insects and animals, physical and sexual violence and worsening of chronic diseases [2]. The epidemiological surveillance of mass gatherings has a preeminent role in the management of potential health risks to the populations involved in the detection of outbreaks and other events of importance to public health. Public health activities during mass gathering also include the prevention and/or minimization of the risk of occurrence of injuries and diseases and maximizing the safety of participants, viewers/assistants, employees, as well as residents [2, 6-8]. The National Festival of Culture occurs every two years and celebrate the cultural diversity of Mozambique with artists from various specialties with emphasis on traditional music and dance, singing, crafts, theater, cinema, fashion and gastronomy. The 9^th^ edition of the Mozambique National Festival of Culture took place in the cities of Beira and Dondo in Sofala Province between August 24 to 28, 2016, involving the participation of approximately 5,000 people from Mozambique and the region comprising of national and international artists, organizers, officials, media professionals and spectators. With an objective to monitor health events, including the timely detection of possible outbreaks during the 9^th^ National Festival of Culture, an epidemiological surveillance system was implemented with the ability to collect data in real time using mobile technologies through the Epi Info™ 7.

## Methods

A real time surveillance system was established for the event to capture information on all the health consultations that occurred in designated fixed points in Beira City and Dondo District, from August 24 to 28, 2016. Beira is the capital of Sofala, the second largest city in the county, is situated in the center of the country with a population of 463.442 inhabitants [9]. In turn, the district of Dondo is located 30km from the city of Beira, with a population of 180,905 inhabitants [9]. The data collection was performed using the electronic forms in EpiInfo ™ version 7 (CDC, Atlanta), installed on tablets which sent data via mobile internet to an electronic database, from 15 fixed health posts set-up at the festival in the two cities where the festival occurs: 10 in Beira and 5 in Dondo. The electronic forms collected information on socio-demographic variables, symptomatology, provisional diagnosis, places of lodging and the health posts. The coordinators of the health posts also input the data into the online system and received a 1 day (8 hour) training on collecting data and the use of tablets. Cases were defined as *"every individual that before seeking care was attending or working at the cultural festival, or that he suffered any damage or accident during or related to the festival, in the period from August 24 to 28."* The monitoring of the data was carried out daily at the fixed health post health posts set-up at the festival using the dynamics of the Incident Command System (ICS) [10] in addition to the onsite monitoring of the coordinators at each site. Data analysis was performed using EpiInfo™ version 7, in an online Dashboard module, using measures of frequency and central tendency

## Results

**Figure 1:**
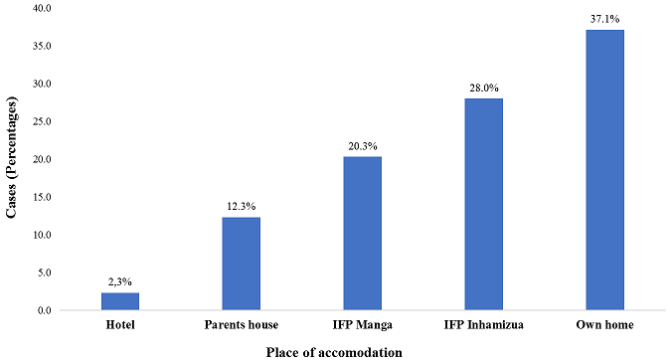
Distribution of symptoms recorded during the festival, from August 24 to 28 (Source: Dashboard, CIOCS/Beira 2016)

**Figure 2:**
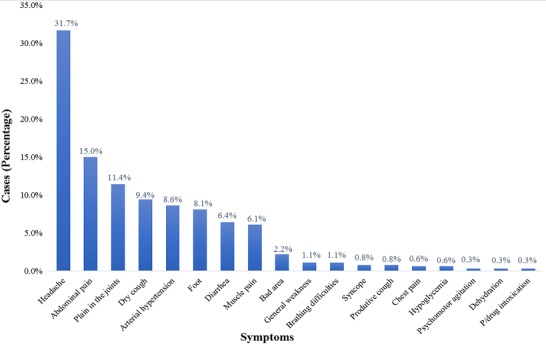
Diagnostic distribution duties during the festival, from August 24 to 28 (Source: Dashboard, CIOCS/Beira 2016)

**Figure 3:**
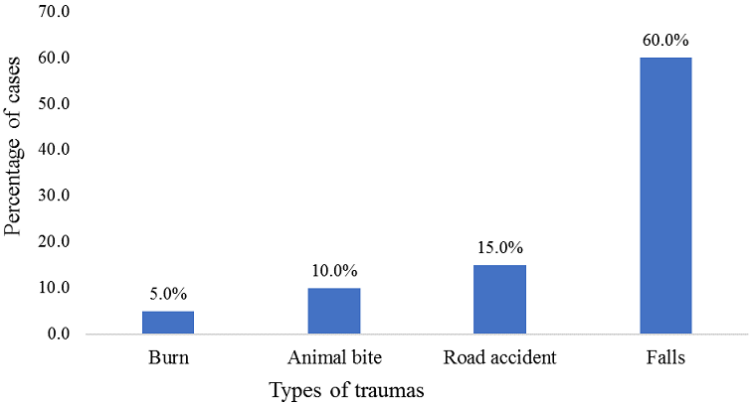
Distribution of visits by trauma (Source: Dashboard, CIOCS/Beira 2016)

**Figure 4:**
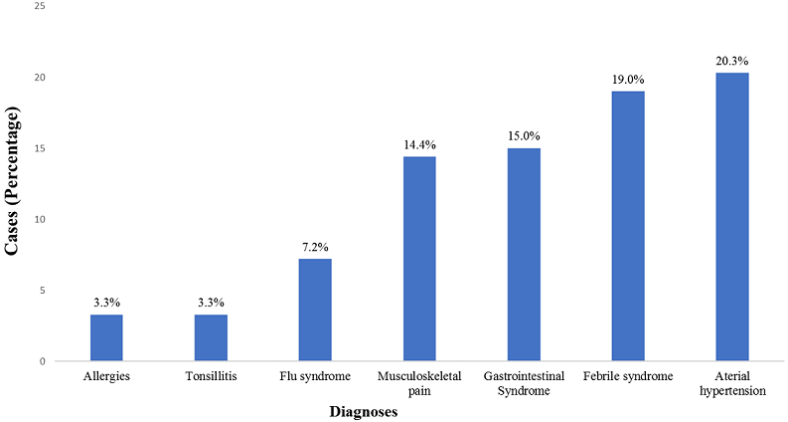
Distribution of health consultations among health posts (Source: Dashboard, CIOCS/Beira 2016)

During the event, a total of 355 individuals sought health care, of which approximately half (52.3%) were females and the majority were artists (62.2%). The age ranged from 4 to 71 years, with a median age of 26 years. Overall, people above 45 years of age sought health care more frequently (30.8%) than other age groups. During the event, the peak of visits occurred on the third day of the festival (Table 1). Beira city registered 309 (87%) patients and the Campo do Ferroviário health post saw over 96 cases (27.0%) during the festival. In the city of Dondo there were 40 (11%) cases attended. Regarding the distribution of cases treated by place of lodging, 130 (37.1%) cases were staying in their own homes, 98 (28%) were staying at the Institute for Training Teachers of Inhamízua, 71 (20.3%) were housed at the Institute for Training Teachers of Manga and 43 (12.3%) in the house of relatives (Figure 1). The clinical symptoms most commonly seen were headache, 114 (31.7%), followed by abdominal pain, 54 (15%), and pain in the joints 41 (11.4%) (Figure 2). Traumas were one of the causes of seeking health consultations, with 20 (100%) cases. Falls were the most common type of trauma with 12 cases (60.0%), followed by traffic accidents with 3 cases (15.0%), and an animal bite with 2 cases (10%) (Figure 3). The main diagnoses issued by health professionals at the health posts during the festival were arterial hypertension with 31 (20.3%) cases, followed by febrile syndrome with 24 (19%) cases, and gastrointestinal pain syndrome with 23 (15%) cases (Figure 4). As to the outcome, the vast majority were discharged (98.3%), 4 (1.2%) were transferred, and 2 (0.6%) abandoned treatment. There was no record of death during the event.

**Table 1:** General characteristics of health consultations during the 9^th^ National Festival of Culture in Mozambique, Beira 2016

Characteristics of health consultations	N	%
***Sex***		
Male	171	47.7
Female	184	52.3
**Age Range**		
5-14	3	7.7
15-24	7	15.4
25-34	62	25.6
35-44	50	20.5
>=45	68	30.8
**Cases sites**		
Beira City	309	87.0
District of Dondo	40	11.0
**Profession/ Classification**		
Artists	211	62.2
Assistants	45	3.2
Protocol	41	2.1
**Day of the festival**		
1^st^ Day	43	12.0
2^nd^ Day	78	22.0
3^rd^ Day	122	34.4
4^th^ Day	77	22.0
5^th^ Day	35	10.0
**Health Posts**		
C. Ferroviário	96	32.3
C. Chigunssura	90	30.3
C. Munhava	67	22.6
C. UC and Arts	12	4.0
House of artists	1	0.3
**Home Location of Participants by Province**		
Sofala	199	56.4
Manica	29	8.2
Inhambane	25	7.1
Nampula	18	5.1
Zambézia	12	3.4
Tete	14	4.0
Cabo Delgado	15	4.2
**Total**	355	

## Discussion

This was the first experience of implementing real time monitoring surveillance system for a mass gathering in the country. The experience was successful and can be used for surveillance during upcoming events. The results indicated that the largest number of visits was recorded on the third day of the event. However, it was expected that the increased demand for health care services were the first and last days, due to the higher concentration of people to participate/attend the ceremonies of the opening and closing of the festival. These findings are similar to those already reported in other studies which found that the greater number of visits between the second and fourth days, a period in which occurs the greatest number of physical activities [11]. The main symptom for seeking health care was a headache, which also has been observed in other similar events [11, 12]. The long environmental exposure of participants can result in dehydration and sunburn, which can cause headaches, since a large part of the activities took place during the day in open fields. Adults above 45 years of age were the highest seekers of health services; complaints were commonly related to hypertension and age, which has already been observed in several studies [13, 14]. The audience for this type of event is composed mainly of adults who participate in the activities of cultural dance, crafts, theatre and cuisine. The fact that medical assistance available in participants' home areas, which may be rural communities that may face limitations and difficulties to access health care, can lead people to take advantage of the health services available during the festival to monitor their state of health.

In the consultations for symptoms related to trauma, falls were prevalent. This fact was expected considering the nature of the event, which involves to a large extent skeletal muscle movement. The main clinical diagnoses were febrile syndrome, acute gastrointestinal and musculoskeletal pain. This pattern of gastrointestinal disorders is often associated with the change in the pattern of food, the sanitation conditions in places of events and compliance with the basic rules of hygiene [12, 15]. The febrile syndrome and musculoskeletal pain are often related to exhaustion, agitation and the noise produced at the event [15-17]. Although the system has been successfully implemented, this analysis has some limitations. The data were entered by local field supervisors and not by the staff responsible for the observation of patients in the health posts, which caused a slight delay in the analysis in real time. The monitoring system was implemented only on the days in which the festival took place, but the concentration of participants at the site of the event began days before and prevailed a few days after the event, in this way the system may have lost some cases and cannot include disease caught at the festival that only showed symptoms after. Initial screening upon arrival for mass gathering events is a critical component and should be considered also for detecting outbreaks.

## Conclusion

The monitoring system worked as planned and benefited from active collaboration with the Mozambican health authorities from different levels. The use of technology to send data directly from the field can be cited as a successful experience for real time monitoring of health outcomes during mass events and can be used during similar events in the future. The Mozambique MoH plans to continue building real time syndromic surveillance systems to monitor health events during mass gatherings.

### What is known about this topic

- Mass gathering events can impact public health due to an increased number of infectious diseases and trauma;
- Public health surveillance during mass gatherings events creates an opportunity to identify health conditions and threats in a timely manner;
- There is an increase use of real time internet-based methods for infectious diseases surveillance during mass gatherings.

### What this study adds

- This is the first article describing the experience using real-time technology to monitor health events during mass gatherings in Mozambique;
- During the Mozambique 9^th^ National Festival the main provisional diagnoses of those who were attended to during the festival was arterial hypertension, followed by febrile syndrome. Falls were the most frequent causes of trauma during the festival;
- Initial screening upon arrival for mass gathering events is a critical component and should be considered also for detecting outbreaks.
